# Editorial: Forage crop improvement for improved livestock production and nutrition

**DOI:** 10.3389/fpls.2022.1082873

**Published:** 2022-12-05

**Authors:** Jorge F. Pereira, Jiyu Zhang, Russell Jessup, Chris S. Jones

**Affiliations:** ^1^ Embrapa Gado de Leite, Juiz de Fora, Brazil; ^2^ State Key Laboratory of Herbage Improvement and Grassland Agro-Ecosystems, College of Pastoral Agriculture Science and Technology, Lanzhou University, Lanzhou, China; ^3^ Department of Soil and Crop Science, Texas A&M University, College Station, TX, United States; ^4^ Feed and Forage Development, International Livestock Research Institute, Nairobi, Kenya

**Keywords:** animal nutrition, forage quality, genome-wide association studies (GWAS), marker assisted selection, plant breeding, omics

We have a major challenge to sustainably grow the production of animal protein in order to feed an additional nearly two billion people by 2050. In addition, in some regions of the world, population is also predicted to be wealthier, which positively impacts consumption of animal protein. Increasing animal production is a multifaceted challenge and one component that needs to be tackled is the production and quality of the forages that they feed on. Improved forages can increase animal production per unit head and may also allow higher grazing intensity (more animals per hectare), leading to lower pressure on grazing land which is the most predominant way of feeding livestock. However, even in locations where animal feeding is based on cut and carry and indoor systems, forages are an important resource.

In order to improve forages for better livestock production, plants need to be bred by combining desired characteristics in one genotype or family. However, forage breeding faces many challenges including variable ploidy levels, different reproductive modes, and the need to evaluate traits that are laborious to assess. Besides, breeding efforts are distributed across hundreds of forage species that need to be adapted to a vast range of systems and environments worldwide.

Conventional forage breeding is a time-consuming process (that needs up to 10 years to develop a cultivar) and is mostly based on traits that are often poorly understood. Here, molecular sciences and biotechnology offer great possibilities to develop better breeding pipelines to pave the way for accelerated and more efficient forage breeding. This Research Topic *“Forage Crop Improvement for Improved Livestock Production and Nutrition”* focused on studies leveraging molecular technologies to capture the opportunities from forage genetic diversity to support the accelerated development of new cultivars. The Research Topic contains 15 original research articles based on 11 legume and grass forage species, where several molecular approaches (genome-wide association studies - GWAS, transcriptomic, proteomic, metabolomic, linkage, and QTL mapping) were used to characterise traits including flowering time, heavy metal stress, nutritional quality, phosphate accumulation, rust resistance, salt tolerance, seed coat permeability, and yield.

The most common forage species studied in this Research Topic is alfalfa (*Medicago sativa*), a high-protein legume forage that is widely used especially in temperate regions. Flowering time in alfalfa, which effects biomass yield and quality, is affected by genetic and environmental factors. Thus, the identification of a marker (gene, protein, or metabolite) associated with flowering time could have important applications in alfalfa breeding. Two papers in this Research Topic report the identification of candidate genes associated with flowering time in alfalfa. Jiang et al. identified 16 and 22 QTL for early and late flowering traits, respectively, and gene expression analysis revealed thousands of up- and down-regulated transcripts. The QTL and gene expression data were integrated, resulting in the detection of seven flowering time gene candidates. He et al. found that geographical origin and breeding status strongly influenced alfalfa flowering time. The researchers combined GWAS and transcriptomic analyses to identify 38 candidate genes and two SNPs, located upstream of one candidate gene, were significantly associated with flowering time. The trait was also studied by Liu et al., but with the intention of understanding the molecular mechanisms underlying variation in nutritional quality. These authors used a multi-omics approach to evaluate chlorophyll, amino acid, and flavonoid content in alfalfa leaves at budding and full-bloom stages. As alfalfa matures the content of chlorophyll, amino acids, and flavonoids in leaves decreases. This was correlated with the up- or down-regulation of specific genes, which may guide future endeavours to obtain high-quality alfalfa. Several alfalfa quality traits, alongside biomass yield and plant height under water-limited environments, were studied by Jiang et al. By combining linkage mapping and RNA-seq analysis, they detected 22 common differentially expressed genes located in nine QTL regions that explained >10% of the phenotypic variation. This knowledge may guide new studies about the effect of drought on alfalfa nutritional quality.

After alfalfa, the forage species with most studies in the Research Topic is *Elymus sibiricus*. Another species from this same genus, *Elymus nutans*, was also studied. Zheng et al. reported the identification of regulatory networks and hub genes related to flowering time in *E. sibiricus*. After identifying accessions varying in booting, heading, and flowering times, transcriptome analyses revealed 72 candidate genes with four of them significantly upregulated in late-flowering accessions. Drought and salt stress appear to activate the flowering regulation pathway, and a SNP found in one of seven hub genes was detected and associated with late flowering. Zhang et al. provided relevant genetic information for the gigantic genome (6.86 Gbp) of *E. sibiricus*. They were interested in laying the foundation for molecular mechanisms of the species adaptation to high altitudes as well as of yield- and seed-related traits. Their results revealed genes associated with low oxygen and strong ultraviolet radiation (important factors in higher altitudes) and a total of 1,825 significant loci associated with 12 agronomic traits. For *Elymus nutans*, Zhou et al. were interested in elucidating the mechanism of seed coat permeability, which controls exchange of substances (water, gas, and nutrients) between the seed embryo and the outside environment, and can affect seed dormancy. The authors identified thiamine and salicylic acid as the key metabolites affecting seed coat permeability and expression of the *PR1* and *PAL* genes correlated with these two metabolites. These metabolites and genes may be important targets for better seed production of *E. nutans*.

The transport of phosphate, zinc and manganese were investigated in three papers of this Research Topic. Wang et al. were interested in investigating the role of the MtPT5 transporter in leaf growth and inorganic phosphate (Pi) accumulation of *Medicago truncatula*. By using expression analysis, complementation of *Arabidopsis* mutants, and overexpressing *MtPT5* in *M. truncatula*, the authors were able to demonstrate that MtPT5 plays important roles in vegetative growth and Pi nutrition. Li et al. focused on the impact of zinc (Zn) and the identification of Zn transporters in *Ceratoides arborescens*, a forage that can be grown in arid and semiarid regions. The *CaMTP* gene was detected in the root transcriptome of *C. arborescens* and complementation of *CaMTP* in a yeast mutant conferred its ability to grow under high Zn conditions. The identification of *CaMTP* significantly contributes to elucidating the molecular mechanisms underlying Zn toxicity in *C. arborescens*. Metal stress, caused by manganese (Mn), in the forage legume *Stylosanthes guianensis*, was investigated by Zou et al., who employed gene expression analyses to identify the *SgNramp1* gene. Higher gene expression was correlated with enhanced Mn uptake in *S. guianensis* and heterologous expression of *SgNramp1* complemented the phenotype of a Mn uptake-defective yeast. Taken together, these results pave the way for better understanding of the response to Mn stress in forages.

The other five papers in this Research Topic report on investigations of the response to biotic and abiotic stresses as well as molecular mechanisms involved in quality traits and better degradation during rumen fermentation. Zhao et al. used transcriptome analyses to elucidate the resistance mechanisms and candidate genes associated with yellow rust resistance in triticale. The results revealed a range of molecular defence strategies in triticale when infected by yellow rust, and this list of candidate genes represents a resource for yellow rust resistance breeding. Transcriptome analysis was also employed by Li et al. to understand the molecular response of *Bromus inermis* to salt stress. Thousands of differentially expressed genes were obtained from leaves and roots and, after enrichment analyses, a set of key genes involved in salinity adaptation was detected. This list of genes now needs to be carefully studied in order to further understand the salinity adaptation mechanism in *B. inermis*. For fodder quality, Bageel et al. were interested in understanding the environmental conditions promoting or inhibiting mimosine and tannin biosynthesis, two secondary metabolites of giant leucaena (*Leucaena leucocephala* subsp. *glabrata*) that are undesirable in higher concentrations. Different environmental conditions (salinity, pH and nitrogen availability) had varying effects on mimosine and tannin biosynthesis. Using transcriptome analyses, the authors were able to identify two genes for mimosine metabolism and five for tannin biosynthesis. In general terms, mimosine content and the expression of mimosine synthase in the foliage were correlated and the increase in tannin production seemed to be affected by the expression of three tannin biosynthesis genes. These results may encourage the design of molecular approaches that inhibit the expression of these genes in order to reduce mimosine and tannins in giant leucaena foliage. The quality of forages not only involves the reduction of undesirable metabolites but also the increase of desirable ones. Zhang et al. studied crude protein content in order to understand the expression of this quality trait in kenaf (*Hibiscus cannabinus*) leaves. Proteomic and transcriptomic approaches revealed four genes associated with high crude protein content in kenaf leaves. These genes, and the proteins that they encode, need to be functionally characterized to help breeding high crude protein kenaf varieties. Better quality forages may also be obtained by evaluating their ruminal fermentation process or even by selecting materials that generate lower methane production. Yi et al. evaluated a rice *BC15* gene mutant, which influences cell wall composition. The authors observed that the *BC15* mutant had enhanced degradation at the early stage of rumen fermentation, which led to decreased methane production. On the other hand, the *BC15* mutant showed lower fibre degradation and cellulolytic fungi population indicating that its straw is more resistant for microbial degradation. It remains to be seen if other forage species with the *BC* (*Brittle Culm*) mutation may have similar characteristics.

This Research Topic demonstrates how the use of molecular sciences and biotechnology can improve the understanding of molecular mechanisms underlying important traits in forage species. The use of this information can be used to accelerate forage breeding in order to sustainably increase the production of animal protein and improve food security. We welcome everyone to explore the 15 research papers for deeper analysis of their methodologies and findings.

## Author contributions

CJ and JP drafted the manuscript. JZ and RJ checked the manuscript and suggested modifications. All authors contributed to the Editorial and approved the submitted version.

